# Self-reported postpartum morbidity: prevalence and determinants among women in Marrakesh, Morocco

**DOI:** 10.1186/s12978-015-0066-z

**Published:** 2015-08-25

**Authors:** Noureddine Elkhoudri, Hakima Amor, Abdellatif Baali

**Affiliations:** Laboratory of Human Ecology, Faculty of Science Semlalia, Cadi Ayyad Marrakesh University, Avenue Prince Moulay Abdellah, BP 2390 Marrakech, Morocco

## Abstract

**Background:**

Maternal mortality is a public health problem particularly in developing countries. This is mainly related to maternal morbidity, especially during the post-partum period (Haemorrhage, infections…). In Morocco, little is known about maternal morbidity within the population. The aim of this study is to determine the prevalence of self-reported postpartum morbidity and grasp its determinants.

**Methods:**

This descriptive and analytic cross-sectional survey was carried out in six health centers drawn randomly in Marrakesh, Morocco. A total of 1,029 women of reproductive age (15–49) giving birth in the year preceding the survey were enrolled. Women were examined in these health centers during the study period.

A questionnaire gathered information about socio-demographic, health and reported postpartum morbidity.

Bivariate and multiple analyses were used to identify associated factors with the self-reported postpartum morbidity. Statistical significance was set at *p* < 0.05.

**Results:**

The self-reported postpartum morbidity prevalence was 13.1 % while haemorrhage, pregnancy-induced hypertension and fever were the main complications: 71.92 %; 12.18 % and 10.64 % respectively.

According to the multiple logistic regression model, the illiteracy among women and the number of pregnancies greater than 3 determine independently this morbidity (OR = 1.24; CI 95 %: 1.09–1.54; and OR = 1.69; CI 95 %:1.04–2.70 respectively).

**Conclusion:**

Reducing female illiteracy and fertility will help the fight against postpartum maternal morbidity, which is critical to the wellbeing of women and their infants.

## Background

Maternal mortality is a public health problem. Every day, approximately 800 women die from causes related to pregnancy and childbirth. WHO defines maternal death as “the death of a woman while pregnant or within 42 days of termination of pregnancy, irrespective of the duration and the site of the pregnancy, from any cause related to or aggravated by the pregnancy or its management but not from accidental or incidental causes” [1].

Almost all maternal deaths (99 %) occur in developing countries [1]. In Morocco, although the prevalence of maternal mortality has been in a sharp decline - it was 601 per 100 000 live births in 1982; 384 in 1990; to 262 in 2000 and 124 per 100 000 births in 2008. It is still high compared to some Arab countries in the same socio-economic and demographic conditions, such as Tunisia, Jordan and Lebanon where it reaches respectively 36; 35 and 24 deaths per 100,000 births [2].

However, maternal mortality is only the tip of the iceberg. The hidden part is maternal morbidity, because 80 % of maternal deaths are related to it after all [3, 4]. Sixty percent of maternal deaths occur in the postpartum period [5]. Postpartum haemorrhage is the leading cause of maternal mortality at a worldwide scale [6, 7]. Therefore the associated variables of these most common maternal morbidities are alternative indicators to those specific to maternal mortality [8].

Age, obstetric history, multiparity and nutritional deficiencies such as anaemia are among the risk factors for maternal morbidity [9]. These factors, outlined in several studies on maternal morbidity, showed a particular interest in pathogenic and biomedical risk factors at the expense of socio-demographic and behavioral ones on the basis that it is considered medical rather than epidemiological or social problem [10].

In Morocco, most studies are based on information from clinics or hospitals, but, because a large proportion of Moroccan women typically does not attend postnatal care (31.1 % in Moroccan urban areas) [11], results do not reflect the true magnitude of the problem [12–14]. Therefore, our aim is to study the self-reported postpartum morbidity (SRPPM) during the last pregnancies from a sample of Moroccan women living in Marrakesh and grasp its determinants.

## Subjects and methods

### Context

In Morocco, antenatal and postnatal care is provided in all 2,689 public health centers and in the private sector. Antenatal coverage is about 77 % (92 % in urban areas and 63 % in rural areas). Most deliveries take place in public hospitals (51.5 %) or in the 606 first-level public delivery houses managed by midwives and general practitioners (12 %); 26.8 % of women deliver at home. Only 9.2 % of women deliver in private hospitals [15].

### Study site

Marrakesh is situated in the center of Morocco and has a population of 1,148,000 habitants. Maternal health services in the city are offered by 63 public health centers with six public ‘delivery houses’ (peripheral primary level), eight private clinics, 34 private general physicians and 13 private laboratories. In the case where complications ensue, women are referred to the secondary-level regional hospital or the tertiary-level university hospital in Marrakesh [15].

### Study design

This is a descriptive and analytic cross-sectional survey. Women of reproductive age (15–49), giving birth during the year preceding the survey were enrolled. They were examined in health centers for medical consultation or children vaccination during the study period (January–October 2014). Ninety-three percent of eligible patients agreed to participate in the study.

The cluster random sampling was used to select six health centers from the list of health centers in Marrakech. Subsequently, 172 women were included from the patients examined in each selected health centre, giving a total of 1,029 women. All participants provided consent before participating in the survey. The information was collected anonymously and confidentially.

A pretested questionnaire makes it possible to collect socio-demographic and health information that might be associated with SRPPM.

The variables used in this study were: maternal age, education level, couple’s occupations, medical insurance, number of pregnancies, failed pregnancy, antenatal (ANC) and postnatal care (PNC), place of birth of the last pregnancy, mode of delivery, and SRPPM.

### Data analysis

A descriptive analysis was performed using means, standard deviations (SD) and proportions as appropriate. To estimate the significance of the differences observed between the means, the Student’s *t*-test was used and the Chi-square test was used for categorical variables. The multivariate analysis which allows the elimination of the confounding factors and entering the weight of the associated variables with the SRPPM in the bivariate analysis (*p* < 0.2), was used to identify factors independently associated with SRPPM. Associations were measured in Odds ratio (OR) with 95 % confidence intervals (95 % CI). The statistical significance was set at *p* < 0.05 and Statistical Package for the Social Sciences (SPSS for Windows, version 10.1) was used for all statistical analysis.

## Results

### Socio-demographic and health characteristics of studied women

The age during the last childbearing ranges from 15 to 49 years, with an average of 28.2 years (SD = 6.4). Women aged over 35 years in their last childbearing representing 13.9 %. The illiteracy rate among women was 25.1 %. For the literate, 29 % attended the primary-school level, 37.3 % the secondary-school level and only 8.6 % reached higher levels in their studies. For their spouses on the other hand, the illiteracy rate is only 15 %, with 32.1 % reaching the primary level, 37.7 % the secondary level and 15.3 % reaching higher levels of studies. Another fact is that female employment is very low since 91.5 % of them were jobless and only 28.6 % had medical insurance. As for their spouses, all of them were involved in professional activities with 74.1 % being workers, craftsmen, employees, drivers, shopkeepers etc. (grouped in the socio-professional category 1: SPC1), and 25.9 % were either state functionaries or had liberal professions (grouped into socio-professional category 2: SPC2).

Furthermore, the number of pregnancies per woman ranges from one to 13 with an average of 2.2 (SD = 1.5), and the live births from one to eight with an average of 2.17 (SD = 1.16). The observed difference between the number of pregnancies and the number of live births was statistically significant (t = 5.6; *p* < 0.05). It is attributed to the number of failed pregnancies which varies from zero to six with an average of 0.26 (SD = 0.60).

The antenatal care coverage (ANC) was of 82.2 %. During those medical consultations, the majority of women received measurements for weight (94.5 %), height (94.7 %), blood pressure (93.4 %) and blood analysis (85.6 %), iron supplementation (79 %). However, only 41.4 % of them received urinalysis (testing albumin and sugar levels). These medical services were received free of charge by the public health centers, while the private sector is sought mainly for ultrasounds and blood tests.

Almost all women (97.6 %) delivered in a health facility, with 81 % in public hospitals and 16.7 % in the private clinics. Fifteen percent of the enrolled women had caesarean delivery. The proportion of women who attended a postnatal consultation was 30.1 %. Lack of complications (68.6 %), lack of information (17.2 %), financial difficulties (10.9 %), and bad experiences at hospitals (3.3 %) were the main reasons reported for not attending these consultations.

### Self reported postpartum morbidity prevalence and determinants

**Fig. 1:**
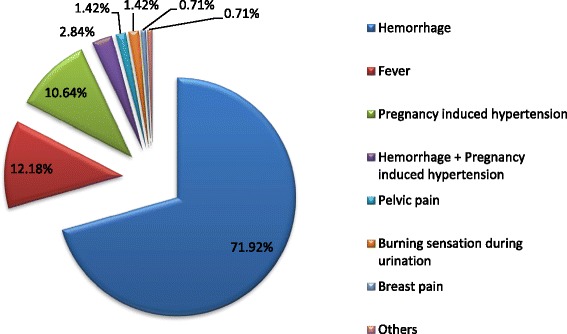
Women’s reported postpartum morbidities

SRPPM prevalence was of 13.1 %. Haemorrhages, fever and pregnancy-induced hypertension were the main complications: 71.92; 12.18 and 10.64 % respectively (Fig. 1). In bivariate analysis, female’s age at the last childbearing greater than 35 years, illiteracy, women having more than three pregnancies, failed pregnancies (if any) and delivery in a public hospital were related to the SRPPM Table 1. According to the multiple logistic regression model, the two factors that determine significantly and independently the SRPPM were illiteracy and the number of pregnancies exceeding three. OR = 1.24; CI 95 %: 1.09–1.54; and OR = 1.69; CI 95 %:1.04–2.70 respectively (Table 2).

**Table 1:** Socio-demographic and health variables influencing women’s reported postpartum morbidity

Variable	Modalities	Number	Self reported postpartum morbidity		*p*
			Present% (n)	Absent% (n)	
Woman’s age at the last childbearing	15–24	371	9.7 (36)	90.3 (335)	**0.02**
	25–34	514	14.2 (73)	85.8 (441)	
	35–49	144	18.1 (26)	81.9 (118)	
Education level	Illiterate	258	18.2 (47)	81.8 (211)	**0.01**
	Primary	298	13.1 (39)	86.9 (259)	
	Secondary and more	473	10.4 (49)	89.6 (424)	
Women occupation	Yes	87	10.3 (9)	89.7 (78)	0.42
	No	942	13.4 (126)	86.6 (816)	
SPC of the spouse	SPC1	769	13.0 (100)	87,0 (669)	0.85
	SPC2	260	13.5 (35)	86.5 (225)	
Insurance health coverage	Yes	294	13.3 (39)	86.7 (255)	0.93
	No	735	13.1 (96)	86.9 (639)	
The number of pregnancies	≤3	593	9.4 (56)	90.6(537)	**0.00005**
	>3	436	18.1 (79)	81.9(357)	
Failed pregnancy	Yes	206	18.4 (38)	81.6(168)	**0.01**
	No	823	11.8 (97)	88.2(726)	
Place of birth	Public	833	14.2 (118)	85.8(715)	**0.02**
	Private	171	7.6 (13)	92.4(158)	
The mode of delivery	Vaginal	875	13.8 (121)	86.2(754)	0.10
	Caesarean	154	9.1 (14)	90.9(140)	
Antenatal care	Yes	846	12.4 (105)	87.6(741)	0.15
	No	183	16.4 (30)	83.6(153)	
Post-natal care	Yes	310	12.9 (40)	87.1(270)	0.89
	No	719	13.2 (95)	86.8(624)	

The associations between different studied variables and reported maternal morbidity. It is a bivariate analysis. When *p* is less than 0.05 it is written in bold

**Table 2:** Variables influencing the SRPPM according to the multiple logistic regression model

Variables	Modalities	O.R	95 % CI	
Woman age at childbearing	(15–24)	1.26	0.65	2.43
	(25–34)	1.03	0.60	1.75
	**[(35–49)]**	–	–	–
Education level	Illiterate	1.24*	1.09	1.54
	Primary	1.20	0.76	1.91
	**[Secondary and more]**	–	–	–
Number of pregnancies	>3	1.69*	1.04	2.70
	**[≤3]**	–	–	–
Failed pregnancy	**[Yes]**	1.28	0.81	2.04
	No	–	–	–
Place of birth	Public hospital	1.75	0.95	3.23
	**[Private clinic]**	–	–	–

**[]**: Reference modality; *: *p* < 0.05, *O.R* Odds ratio, *CI* Confidence interval

Footnote: All effect estimates were adjusted for the other variable listed in the table

Displays the results of the multivariate analysis. The reference category is in brackets. When the association is significant, the OR is bold

## Discussion

Information on postpartum morbidity in developing countries is limited and, when available, usually describes the type of medical conditions diagnosed at the hospital level [15]. This study is one of the few surveys conducted in Morocco studying SRPPM within the population.

Our results show that 13.1 % of women expressed at least one postpartum problem. The percentage was close to the one recorded in Marrakesh in 2008 which is 10.8 % [16]. However the rate of postpartum haemorrhages (9.91 %) was much higher than the one recorded in 2008 which is 1.5 % [16].

The rates of postpartum haemorrhage are incomparable since the amount of blood loss is difficult to estimate [11]. The rate of fever was 1.5 %, the one recorded in 1999 [11] and 2010 [16] were 3.3 and 2.2 % respectively. Pregnancy-induced hypertension is more common (1.65 %) in comparison with other studies carried out in Marrakesh (0.2 %) [16].

Nevertheless, a limitation of the present study is the wide variation of information reported by women based on socio-cultural and medical context. Hence, it is unclear whether the SRPPM represent the magnitude of the genuine morbidity. Thus, to understand the gap between the reported morbidity and the one defined medically, studies should be conducted to assess the validity of the information reported by women [12].

However, estimates of self-assessed morbidity prevalence are generally more specific than sensitive [15]. Underestimation or overestimation by women tends to be influenced by age, level of education, and specificity of clinical symptoms [15]. In industrialized countries, greater value is given to womens’ self-reports of complaints, and responding to these complaints is considered more important than measuring the incidence of true postpartum morbidity [2].

But in non-industrialized countries such as Morocco, we noted a lack of or in-complete information due to poor documentation about self reported postpartum morbidity [15].

Also, we found that the amount of women in our study population who attend a postnatal clinic is low (30.1 %), and it is also low nationally (22 %) [11, 17]. Our study has brought clear supporting arguments to increase this percentage given the important role of the PNC.

Furthermore, some associations of socio-demographic and health variables with the SRPPM were observed including the woman’s age at the last childbearing, education level, number of pregnancies, failed pregnancies and place of birth.

The age at childbearing over 35 years increases several maternal complications like pregnancy-induced hypertension, dystocia and haemorrhage [18]. In this study, 13.9 % of the participants were found to be 35 and above during their last pregnancy. Retreat from marriage has moved from 17.3 years old in 1960 to 26.6 years old in 2010, can explain partly this finding [19].

Similarly, women who had been pregnant more than three times reported significantly more postpartum complications. This can be attributed to the deeper impact of multiple pregnancies, which causes the exhaustion of the uterine muscle promoting more complications; particularly haemorrhage [18].

Furthermore, the average number of pregnancies (2.17) is lower than the one recorded in 1998 (2.5) [17]. This further shows the continuing decline in fertility in Marrakesh. The use of contraceptive methods is increasing thanks to the national family planning program that provides Moroccan couples a range of free contraceptive methods in public health centres [11].

Illiteracy has been found to be another important determinant strongly associated with the SRPPM. This has been confirmed by other studies conducted in Marrakesh and its regions [16]. In fact, the education level is an important determinant of reproductive and health behaviour [11]. Illiteracy is higher among women than their spouses (*p* < 0.01). This difference is due to socio-cultural factors related to gender and also to disadvantaged socio-economic conditions that hinder access to more advanced levels even for males. The Moroccan educational system has changed and improved significantly over the past two decades through an explicit commitment to ensure compulsory education for all. However, more progress is still to be achieved for a more balanced distribution of education between Moroccans, including the fight against illiteracy and dropouts [11].

In addition, failed pregnancy during the reproductive life is lower than the one found in 1999 (26 %) [11]. It is associated with SRPPM in subsequent pregnancies which was a result also confirmed [16].

Our study shows that the delivery rate in a health facility (97.6 %) is more frequent than at the national scale (90.7 %). Also women who gave birth in a public hospital reported more postpartum complications than those who gave birth in private clinic. Moroccan public hospitals suffer from a shortage of health workers failing to meet the needs of the population [11].

Finally, adjusting on other significantly associated variables in bivariate analysis, and through the multivariate analysis, only illiteracy and over three pregnancies determine the SRPPM independently.

## Conclusion

While reading the results, the SRPPM prevalence is found to be 13.1 %. Haemorrhage, pregnancy-induced hypertension and fever are the major complications. Through bivariate analysis, it is observed that several socio-demographic and health variables determine these reported complications, namely the mother’s age at her last childbearing being above 35, women having more than three pregnancies, illiteracy, failed pregnancy and delivery in a public hospital. In the multivariate analysis, only illiteracy and the number of pregnancies over three were associated to the SRPPM.

What needs is to put action on the modifiable variables, such as illiteracy and fertility, as they will contribute largely to the reduction of postpartum maternal complications which will directly lead to the decline of maternal mortality.
